# Visual detection platform based on RPA-CRISPR/Cas12a for Klebsiella pneumoniae and Carbapenem-resistant Klebsiella pneumoniae in clinical and food safety settings

**DOI:** 10.3389/fcimb.2026.1817859

**Published:** 2026-05-25

**Authors:** Tuo Ji, Xin Fang, YuZhi Gao, Kun Yu, JiaChen He, Lin Wang, WenJun Zhu, GuanHong Huang, XuZhu Gao

**Affiliations:** 1 Lianyungang Clinical College, Bengbu Medical University & The Second People’s Hospital of Lianyungang, Lianyungang, China; 2 College of Life Sciences, Bengbu Medical University, Bengbu, China; 3 Department of Central Laboratory, The Second People’s Hospital of Lianyungang, Lianyungang, China; 4 Department of Medicine Laboratory, The Second People’s Hospital of Lianyungang, Lianyungang, China

**Keywords:** Recombinase polymerase amplification, CRISPR/Cas12a, Klebsiella pneumoniae, Carbapenem-resistant Klebsiella pneumoniae, point-of-care testing, food safety, visual detection under blue light illumination

## Abstract

**Introduction:**

The rise of Klebsiella pneumoniae (KP) and carbapenem-resistant KP (CRKP) poses grave threats to public health and food safety, creating an urgent demand for rapid point-of-care testing (POCT). Traditional detection methods are limited by laboratory barriers, making them unsuitable for POCT implementation.

**Methods:**

Herein, a one-tube assay integrating recombinant polymerase amplification (RPA) with CRISPR/Cas12a technology was developed for the rapid, sensitive and specific detection of KP and *blaOXA-48*-carrying CRKP. Specific primers targeting the KP-specific rpoB gene and carbapenem-resistance gene *blaOXA-48* were designed, and optimal primer pairs were screened via agarose gel electrophoresis. CrRNA sequences were designed according to RPA amplicons, and the components of the CRISPR/Cas12a reaction were optimized. A two-step reaction system was initially evaluated, followed by the establishment of an integrated one-tube RPA-CRISPR/Cas12a assay. A total of 66 clinical specimens and artificially contaminated food samples were used for method validation, with microbial culture and qPCR as reference methods.

**Results:**

The two-step assay was capable of detecting bacterial suspensions at a concentration of 100 CFU/mL. The one-tube system could be completed within 1 hour at 37 °C. This assay avoided aerosol contamination and allowed visual result readout under blue light. In the validation test, the detection results of the one-tube assay were consistent with those obtained by microbial culture and qPCR.

**Discussion:**

This study constructed a dual-target RPA-CRISPR/Cas12a platform for the visual detection of KP and *blaOXA-48*-positive CRKP under blue light. This assay reduces reliance on sophisticated equipment and professional personnel. It can serve as a promising POCT tool for clinical diagnosis and food safety surveillance, and provides evidence for the timely formulation of rational antimicrobial treatment strategies.

## 1 Introduction

*Klebsiella pneumoniae* (KP) is a Gram-negative bacillus with a wide distribution. It colonizes livestock, contaminates retail meats and vegetables, and often inhabits the human respiratory and intestinal tracts (Davis and Price, 2016) (Tominaga, 2018). When human immunity declines or other adverse conditions occur, KP can cause various infectious diseases, such as pneumonia, urinary tract infections, septicemia, and meningitis (Wyres and Holt, 2018) (Yao et al., 2015). In recent years, the abuse of antibiotics has led to the increasingly prominent problem of drug resistance in KP. Antibiotic-resistant KP has been isolated from a variety of meats, seafood, vegetables and other foods, as well as the environment (Davis and Price, 2016). Among them, Carbapenem-Resistant *Klebsiella pneumoniae* (CRKP) has emerged as a significant global public health threat (Wang et al., 2024), with the resistance rate of KP to carbapenem antibiotics increasing year by year. Despite the absence of carbapenem use in food animals, CRKP persists in them (Bai et al., 2024). According to data from the China Antimicrobial Resistance Surveillance Network (www.chinets.com), the resistance rates of KP to imipenem and meropenem increased from 3.0% in 2005 to 22% in 2024. The high detection rate of CRKP, along with its rapidly rising resistance rate and mortality, represents an urgent challenge in the field of anti-infection, both domestically and internationally. Therefore, rapid and accurate detection of KP and its resistance genes is significant for early diagnosis, infection control, and rational drug use.

Diverse diagnostic methods for KP, including pathogen detection, immunological techniques, and molecular biology approaches. Traditional pathogen detection methods, such as culture-based and antimicrobial susceptibility tests, involve cumbersome procedures, long detection times, and reliance on sophisticated laboratory equipment (Bowers et al., 2016). Culturing, purifying, and identifying pathogens often takes approximately one week, requiring technical expertise with low sensitivity, thus limiting their application in remote and economically underdeveloped areas. With the rapid development of molecular biology techniques, quantitative real-time polymerase chain reaction (qPCR), gene chips, biosensors, and other methods have been applied to pathogen detection. These methods offer high sensitivity and specificity while shortening detection time. However, qPCR technology requires expensive thermal cyclers, making on-site rapid detection outside laboratories unfeasible. Therefore, there is an urgent need for a simple, practical, convenient, rapid, highly sensitive, and specific novel method for KP detection. Previously, our research team performed drug-resistant genotyping on CRKP strains clinically isolated from our hospital (**Supplementary Table 1**). It was found that the detection rate of *blaOXA-48* in this region was higher than that of *blaKPC* and *blaNDM*. It can be concluded that *blaOXA-48* is the main carbapenemase gene of CRKP in this region, with a relatively high prevalence. The detection targeting this gene has practical significance for the surveillance of clinically drug-resistant bacteria in the local area.

Recombinase Polymerase Amplification (RPA) is a highly sensitive and selective isothermal amplification technique that enables efficient amplification of target genes at a constant temperature within a short time (Lobato and O'Sullivan, 2018). This technology eliminates the need for complex temperature cycling devices. It relies on the synergistic action of recombinase, single-strand binding proteins (SSB), and DNA polymerase to rapidly initiate and complete exponential nucleic acid amplification, providing a more convenient and efficient technical solution for point-of-care testing (POCT) (Piepenburg et al., 2006).

Clustered Regularly Interspaced Short Palindromic Repeats (CRISPR) was initially discovered in the bacterial immune system. The CRISPR/Cas12a system comprises two core components: crRNA and CRISPR-Associated Proteins (Cas) (Paul and Montoya, 2020). This system enables crRNA-guided Cas12a to recognize and cleave target DNA, with specificity dependent on a TTTV-containing protospacer adjacent motif (PAM) (V = A, C, or G) and the protospacer (Manghwar et al., 2019). The Cas12a-crRNA complex identifies the target nucleic acid sequence in a sample complementary to the crRNA. Upon successful recognition, Cas12a specifically cleaves the target double-stranded DNA (dsDNA), causing double-strand breaks and activating the trans-cleavage activity of Cas12a, which non-specifically cleaves single-stranded DNA (ssDNA) in the sample.

Based on in-depth research on RPA and CRISPR/Cas, multiple research teams have combined RPA technology with the CRISPR/Cas12a system for molecular detection (Liu et al., 2023; Yang et al., 2024). RPA-CRISPR/Cas12a ingeniously integrates the efficient and sensitive nucleic acid amplification capability of RPA with the specific DNA recognition and trans-cleavage function of CRISPR/Cas12a, enabling highly sensitive molecular detection. RPA-CRISPR/Cas12a not only improves the efficiency of nucleic acid detection but also significantly expands the application scope of RPA technology, showing broad prospects in on-site diagnosis of pathogens, screening of drug resistance genes, and other fields. Recently, a light–controlled RPA–CRISPR/Cas12a system targeting the *rcsA* gene was established for KP detection. However, this approach focused on single–target KP identification without covering carbapenem resistance genes, which limits its application in drug–resistant strain surveillance (Pan et al., 2025).

**Figure 1:**
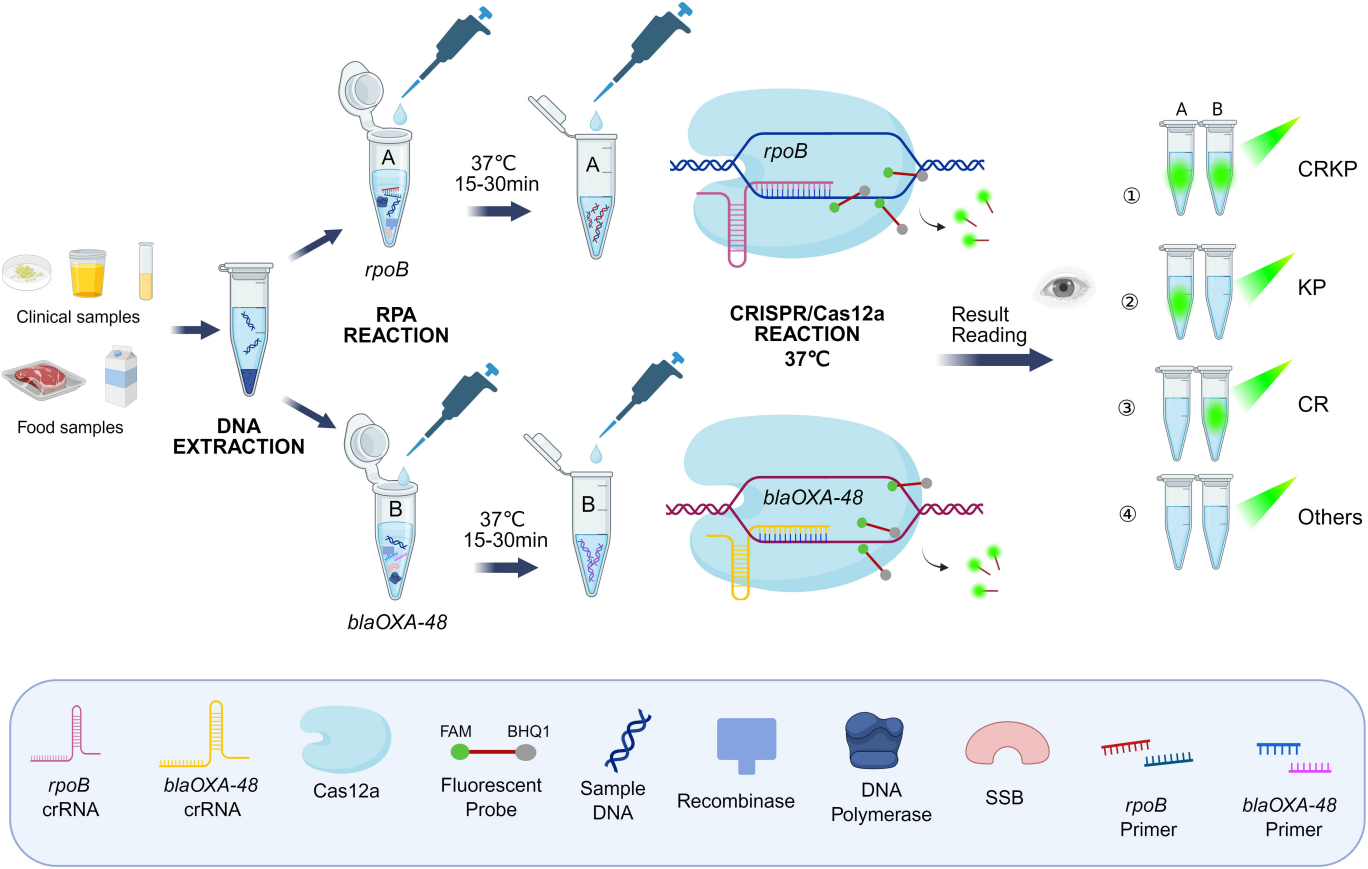
Schematic diagram of the RPA-CRISPR/Cas12a detection system. Genomic DNA is first extracted from the sample. Subsequently, RPA primers targeting *rpoB*, crRNA probes, and other components required for the reaction are added to tube **(A)** In tube **(B)**, RPA primers targeting *blaOXA-48*, crRNA probes, and other reaction components are added. The reaction tubes are incubated in a 37 C water bath, and after incubation, a fluorescence irradiation instrument is used to detect fluorescence signals in the reaction tubes. The criteria for result determination are as follows: fluorescence signals in both tubes **(A, B)** indicate the presence of *bla*OXA-48-positive CRKP; fluorescence exclusively in tube **(A)** (with no signal in tube B) identifies non-*blaOXA-48* KP; fluorescence solely in tube **(B)** (without signal in tube A) indicates *blaOXA-48*-carrying bacteria (non-KP); and the absence of fluorescence in both tubes confirms the sample is free of KP.

This study combines the RPA system with the CRISPR/Cas12a system to detect KP and its *blaOXA-48* drug-resistance gene, enabling synchronous detection of two targets. Further, we develop a single-tube RPA-CRISPR/Cas12a detection method that avoids aerosol contamination and enhances the safety and reliability (**Figure 1**). This system breaks through the dependence on complex sophisticated instruments (e.g., qPCR thermocyclers) and professional technicians, enabling visual detection of amplification products under blue light illumination within a short time. It can serve as a POCT tool for rapid and accurate detection of KP and its drug-resistance genes.

## 2 Methods

### 2.1 Strain sources and genomic DNA extraction

The standard strains used in this study were provided by the Second People’s Hospital of Lianyungang (Jiangsu Province, China) and stored in a -80 C ultra-low-temperature freezer. The microbial strains used in this study were listed as follows: *Klebsiella pneumoniae* (ATCC 10031), *Escherichia coli* (ATCC 25922), *Klebsiella oxytoca* (ATCC51817), *Staphylococcus aureus* (ATCC 25923), *Streptococcus pyogenes* (ATCC 19615), *Salmonella typhi* (ATCC 14028), *Enterobacter aerogenes* (ATCC 13048), *Enterobacter cloacae* (ATCC 13047), *Candida albicans* (ATCC 10231), *Stenotrophomonas maltophilia* (ATCC49130), *Staphylococcus haemolyticus* (ATCC29970), *Staphylococcus epidermidis* (ATCC12228), *Pseudomonas aeruginosa* (ATCC17853), *Haemophilus influenzae* (ATCC49766), *Serratia marcescens* (ATCC14756), *Proteus mirabilis* (ATCC29245). Additionally, strains carrying specific resistance genes were included. blaOXA-48-positive strains: blaOXA-48-type *E. coli*, blaOXA-48-type *P. aeruginosa*, and blaOXA-48-type *E. cloacae*. *Klebsiella pneumoniae* strains harboring other resistance genes: blaNDM-positive *K. pneumoniae* (blaNDM KP), blaKPC-positive *K. pneumoniae* (blaKPC KP), blaCTX-positive *K. pneumoniae* (blaCTX KP), blaTEM-positive *K. pneumoniae* (blaTEM KP), and blaCMY-positive *K. pneumoniae* (blaCMY KP). The standard strain was inoculated onto an agar plate and then incubated in a microbial incubator at 35 C for 24 hours. After colony formation, a single colony was picked and inoculated into 5 mL of culture medium, followed by shaking culture in a shaking incubator at 35 C for 12 hours. Genomic DNA (gDNA) was extracted using the TIANGEN Genomic DNA Extraction Kit, and the gDNA was stored at -20 C for future use. The concentration of gDNA was determined using an ultramicro spectrophotometer. For real samples, DNA extraction was performed using a Quick DNA Extraction Kit (Amp-Future, Jiangsu Province, China), with the specific procedure as follows: the sample was mixed with a rapid nucleic acid release reagent, incubated at 40 C for 10 minutes, and the supernatant was collected for subsequent detection.

To verify the clinical applicability of the RPA-CRISPR/Cas12a assay, a total of 66 clinical specimens were collected between January and June 2025, including 53 sputum samples, 5 urine samples, and 8 bronchoalveolar lavage fluid (BALF) samples. Detailed inclusion and exclusion criteria for these clinical specimens are available in the **Supplementary Materials**.

Meat samples (beef and pork) and milk samples were purchased from local markets in Lianyungang City. Seawater was collected from the Yellow Sea (Lianyungang). The mouse bedding (simulated environmental samples) was purchased from Xiaohe Technology Biotechnology Co., Ltd. These samples were all confirmed to be non-infected with KP by the biochemical culture method.

Beef and pork samples were ground to powder in liquid nitrogen. 100 mg of meat powder was weighed and placed in the bacterial suspension to adjust the final concentration of the bacterial solution to 10^9^ to 10^1^ CFU/mL, followed by thorough mixing. Milk and seawater were mixed with the bacterial solution respectively to prepare samples with a final bacterial concentration of 10^9^ to 10^1^ CFU/mL. Mouse bedding was thoroughly mixed with sterile water, and the supernatant was collected and mixed with the bacterial solution to prepare samples with a final bacterial concentration of 10^9^ to 10^1^ CFU/mL. The total genomic DNA of each sample was extracted using a Quick DNA Extraction Kit (Amp-Future, Jiangsu, China) and stored at -20 C for further study.

### 2.2 RPA primer design and RPA reaction

The inherent gene *rpoB* (NC_016845.1) of KP and the drug-resistant gene *blaOXA-48* (NG_049762.1) of CRKP were downloaded from the National Center for Biotechnology Information NCBI (https://www.ncbi.nlm.nih.gov). Five pairs of RPA primers targeting *rpoB* and *blaOXA-48* genes were designed using Primer Premier 5 software. The RPA primer design followed the following criteria: (1) Primer length of 30–35 nucleotides; (2) Poly-guanine at the 5’-terminus should be avoided as much as possible; (3) Excessive aggregation of purines or pyrimidines in primers should be prevented; (4) GC content of primers between 30% and 70% to avoid secondary structure formation; (5) Amplicon length of 80–500 bp.

The designed primers were validated for specificity using primer-BLAST and subsequently synthesized by General Biosystems Co., Ltd (Anhui, China). The RPA reaction was performed using the Basic DNA Amplification Kit (Amp-Future, Jiangsu, China), according to the manufacturer’s instructions (Ji et al., 2023). The RPA reaction system totaled 50 μL, in which 29.4 μL of reaction buffer, 2.4 μL of 10 μM forward primer, 2.4 μL of 10 μM reverse primer, 13.2 μL of genomic template (100 ng/μL), and 2.5 μL of 280 nM magnesium acetate solution (MgOAc) were added to the reaction tube containing enzyme components. The solution in the reaction tube was thoroughly mixed by inverting up and down, followed by instantaneous centrifugation. The reaction tube was incubated in a 37 C water bath for 30 minutes. After completion of the RPA reaction, the RPA amplification products were extracted using an equal volume of phenol-chloroform-isoamyl alcohol (25:24:1) mixture. Following centrifugation at 12,000 rpm for 5 minutes, the supernatant was collected, mixed with an appropriate amount of DNA loading buffer, and separated by 2% agarose gel electrophoresis (100V, 30min). The RPA reaction products were observed using an ultraviolet gel imaging system. The experiment was performed in triplicate.

### 2.3 crRNA and probe design

Nineteen to twenty-four bases adjacent to the TTTN sequence on the optimal amplicon were selected as the crRNA complementary sequence. crRNA sequences for *rpoB* and *blaOXA-48* genes were designed using the CRISPR RGEN Tools website (http://www.rgenome.net/cas-designer/). The crRNA sequences were retrieved and aligned by BLAST to ensure their specificity. After activation, CRISPR/Cas12a exhibits the property of non-specifically cleaving single-stranded DNA. The short probe sequence can be recognized and cleaved by activated Cas12a, and it can effectively avoid non-specific binding with target sequences or RPA primers, thereby ensuring the detection specificity. Thus, the universal probe sequence was FAM-TTATT-BHQ1 (Fu et al., 2025). Both crRNA and the probe were synthesized by General Biol Co., Ltd.

### 2.4 Construction of two-step RPA-CRISPR/Cas12a reaction system

After RPA amplification under optimal reaction conditions, the CRISPR/Cas12a reaction was performed. The CRISPR/Cas12a reaction system was as follows: 14.5 μL H_2_O, 2 μL 10× Reaction buffer, 0.5 μL Cas12a (2 μM), 2 μL crRNA (10 μM), 1 μL ssDNA (10 μM), and 1 μL of RPA product. In this study, a qPCR instrument was used to collect fluorescence signals, continuously monitoring at 37 C for 60 minutes with data acquisition twice per second.

A series of concentration gradient experiments was conducted to optimize the concentrations of each component in the CRISPR/Cas12a reaction system. First, while keeping other components at constant concentrations, five concentration gradients of single-stranded DNA solutions (500 nM, 50 nM, 5 nM, 0.5 nM, 0.05 nM) were prepared to construct RPA-CRISPR/Cas12a detection systems, respectively. Subsequently, Cas12a protein solutions at concentrations of 100 nM, 50 nM, 25 nM, 12.5 nM, and 6.25 nM were prepared under consistent conditions for other components, followed by the construction of corresponding detection systems. Finally, crRNA solutions with concentrations of 500 nM, 50 nM, 5 nM, 0.5 nM, and 0.05 nM were prepared while maintaining other components unchanged to establish RPA-CRISPR/Cas12a detection systems. The reaction tubes were placed in a qPCR instrument and incubated at 37 C, with fluorescence signals collected twice per minute to monitor real-time changes in fluorescence intensity. After the reaction, the tubes were placed under a fluorescence illuminator (blue light mode, wavelength 460–480 nm) for visual observation of fluorescence signal changes under blue light illumination.

### 2.5 Specificity and sensitivity assessment of the two-step RPA-CRISPR/Cas12a system

To evaluate the specificity of the two-step RPA-CRISPR/Cas12a system, this assay was applied to test other clinically prevalent standard strains and KP harboring other resistance genes, following the previously described detection protocol.

To determine the sensitivity of the two-step RPA-CRISPR/Cas12a system for detecting bacterial suspensions, we prepared serial dilutions of *Klebsiella pneumoniae* and carbapenem-resistant *Klebsiella pneumoniae* carrying *blaOXA48*, with concentrations ranging from 10^8^ to 10^0^ CFU/mL. The two-step RPA-CRISPR/Cas12a reactions were performed on bacterial suspensions of different concentrations. After the reactions, fluorescence intensity was measured and fluorescent signals was observed.

### 2.6 Construction of one-tube RPA-CRISPR/Cas12a system

To adapt this system for application, we designed a one-tube RPA-CRISPR/Cas12a system to further prevent aerosol contamination caused by tube opening. This system achieves temporary separation of reaction components through liquid stratification and surface tension, without the need for additional physical barriers. First, RPA primers, genomic DNA, and magnesium acetate were added to the bottom of the RPA reaction tube. Subsequently, the specific crRNA and fluorescent reporter probe required for the CRISPR/Cas12a system were slowly added along the tube wall. Finally, the Cas12a protein and buffer were added to the inner side of the tube cap. After tightly closing the tube cap, the reaction tube was kept upright; at this point, the Cas12a protein was stably attached to the inner side of the tube cap due to surface tension. The reaction tube was incubated in a 37 C water bath for 30 minutes, then removed, inverted to mix thoroughly, and incubated for an additional 30 minutes before visualizing fluorescence under a blue light illuminator.

To determine the detection limit of the one-tube RPA-CRISPR/Cas12a system, solutions of KP and *blaOXA48*-positive CRKP were prepared at serial dilutions of 10^8^ to 10^0^ CFU/mL. One-tube reactions were performed on these dilutions, followed by fluorescence intensity measurement. Reaction tubes were visualized under a fluorescence illuminator for visual detection of fluorescence signals.

### 2.7 Application validation of one-tube RPA-CRISPR/Cas12a system

To evaluate the performance of the one-tube RPA-CRISPR/Cas12a system in clinical samples, 66 clinical specimens (S1-S66) were collected (**Supplementary Table 2**). Among them, 26 samples were identified as KP-positive by microbial culture (S1-S16, S49-S58), and 6 of these were further confirmed as *blaOXA48*-positive CRKP by qPCR (S1, S2, S49, S52, S53, S56). Forty-eight sets of reaction tubes were prepared, with each set containing two tubes: Tube A with *rpoB* primers and crRNA, and Tube B with *blaOXA-48* primers and crRNA. Genomic DNA from clinical samples was added to both tubes for one-tube RPA-CRISPR/Cas12a reactions. After incubation, tubes were visualized under a fluorescence illuminator for visual detection of fluorescence signals under blue light illumination.

To evaluate the performance of the one-tube RPA⁃CRISPR/Cas12a system for detecting meat samples, we collected 30 common meat samples (including 20 pork samples and 10 beef samples, numbered M1-M30). KP and CRKP were added to meat samples to prepare artificially contaminated samples. Among them, 10 samples were positive samples artificially contaminated with KP (M1-M10, where M1-M6 were contaminated with ordinary Klebsiella pneumoniae, and M7-M10 were contaminated with CRKP carrying the *blaOXA-48* gene), and 20 samples were uncontaminated negative control samples (M11-M30). None of the samples were infected with KP and CRKP before artificial contamination. After pretreating the meat samples, the extracted genomes were added to tubes A and B respectively for the one-tube RPA⁃CRISPR/Cas12a reaction. After the reaction was completed, the reaction tubes were placed in a fluorescence illuminator, and the fluorescence signals were observed with the naked eye under blue light illumination. The processing methods for artificially contaminated milk samples and mouse bedding samples were as described above.

## 3 Results

### 3.1 RPA primer screening and RPA reaction condition optimization

**Figure 2:**
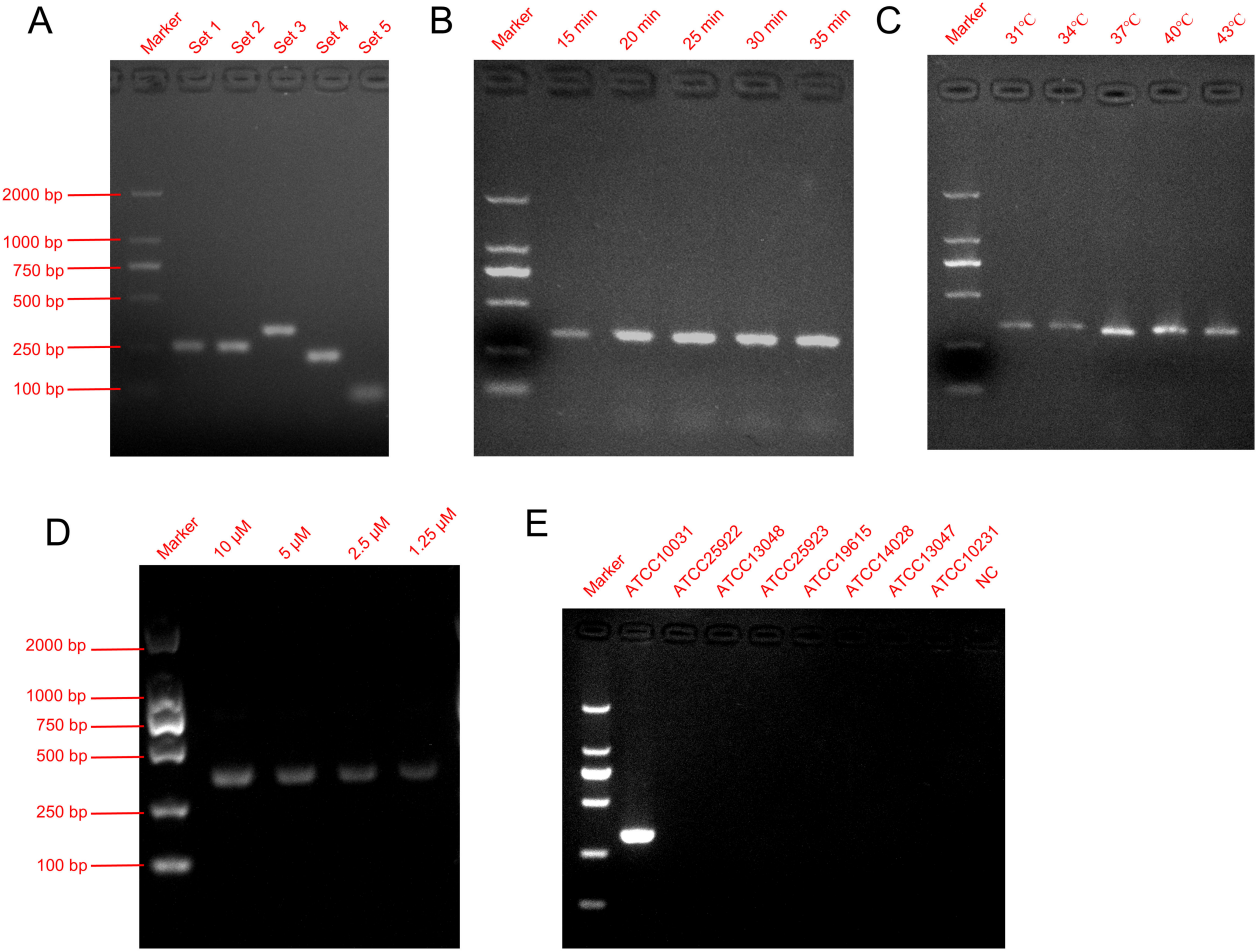
Primer screening for *rpoB* with basic RPA assay. **(A)** RPA primer screening results for the *rpoB* gene. **(B)** Optimization of RPA reaction time for the *rpoB* gene. **(C)** Optimization of RPA reaction temperature for the *rpoB* gene. **(D)** Optimization of primer concentrations for the *rpoB* gene. **(E)** Specificity evaluation of RPA reactions for the *rpoB* gene. Agarose gel electrophoresis was performed using a 2% gel at 100V for 30min.

**Figure 3:**
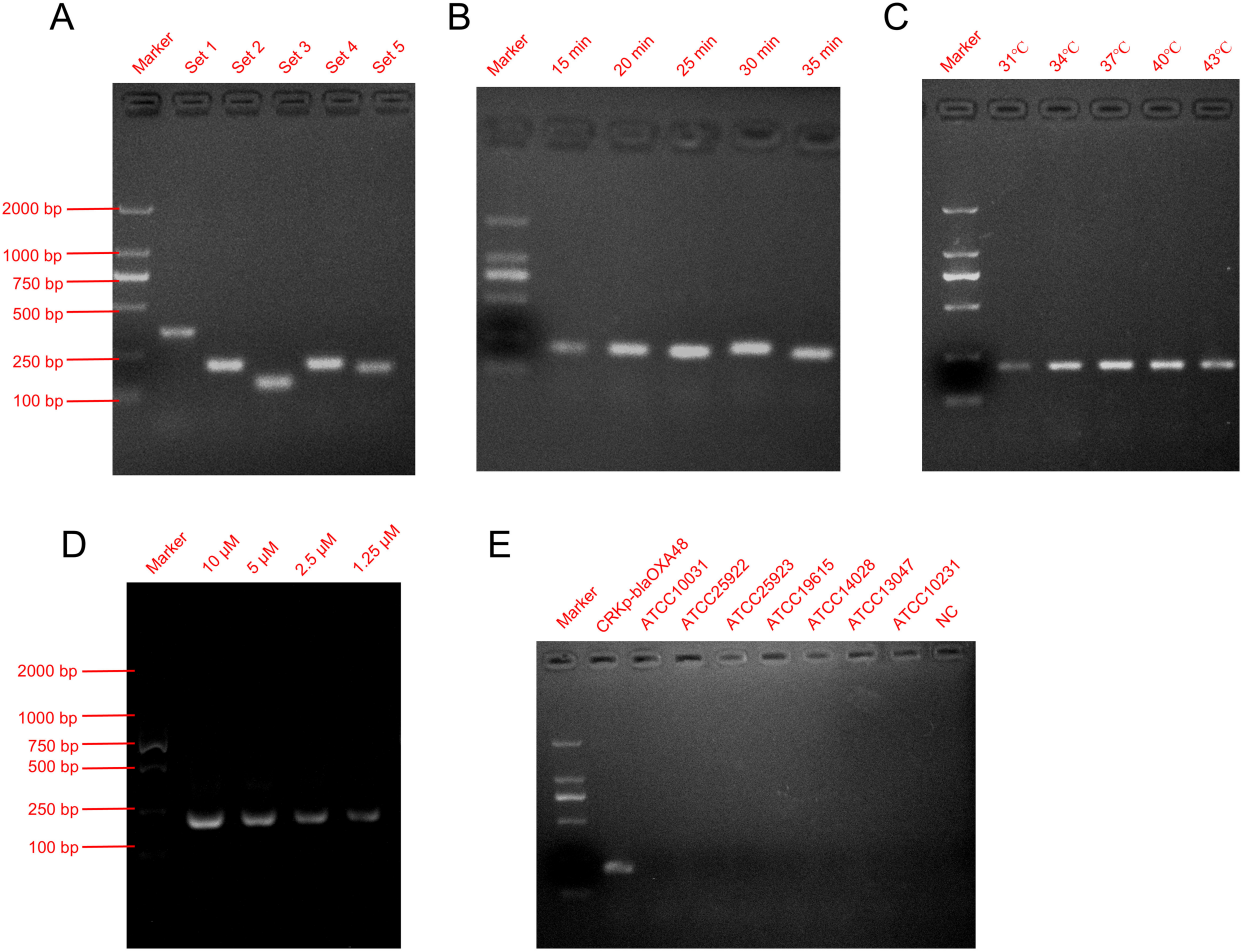
Primer screening for *blaOXA-48* with basic RPA assay. **(A)** RPA primer screening results for the *blaOXA-48* gene. **(B)** Optimization of RPA reaction time for the *blaOXA-48* gene. **(C)** Optimization of RPA reaction temperature for the *blaOXA-48* gene. **(D)** Optimization of primer concentrations for the *blaOXA-48* gene. **(E)** Specificity evaluation of RPA reactions for the *blaOXA-48* gene. Agarose gel electrophoresis was performed using a 2% gel at 100V for 30 min.

Five sets of RPA primer pairs were designed based on the specific gene sequences of *rpoB* and *blaOXA-48* (**Table 1**), followed by basic RPA reactions for the five candidate primer pairs. Aiming at the problem of low-molecular-weight DNA by-products (i.e., “cross dimers”) during RPA amplification, base mismatch optimization of primer sequences was performed according to the following principles: (1) Introduce base mismatches in regions with four or more consecutive paired bases; (2) The 3 nucleotides near the 3’-terminus should not be replaced; (3) No more than 5 bases should be replaced on each primer; (4) No consecutive 2-base substitutions are allowed; (5) A-G and T-C substitutions are preferred. Sequence alignment is shown in **Supplementary Figure 1**. The optimized primer pairs successfully amplified target fragments consistent with the expected sizes. Among them, primer pair 3 for the *rpoB* gene and primer pair 2 for the *blaOXA-48* gene showed the brightest and clearest target bands (**Figures 2A**, **3A**, respectively) with no visible primer dimers under ultraviolet light in a gel imaging system. The gray level value statistics are shown in **Supplementary Figure 2**. Therefore, *rpoB* primer pair 3 and *blaOXA-48* primer pair 2 were selected for subsequent experiments.

**Table 1:** RPA primer sequence.

Set number	Name	Primer sequence (5' – 3')	Amplicon size (bp)
rpoB-Set 1	rpoB-F1	TAAGATGGCGGGTCGTCACGGTAACAAGGG	84
	rpoB-R1	GTACCGTTAGCATCGTGCGGCATATCTTCG	
rpoB-Set 2	rpoB-F2	CGACGGTGCGAAAGAAGCTGAAATCAAAGA	216
	rpoB-R2	AGCGGCTGCTGAGTAACCAGGCTGTAAGAA	
rpoB-Set 3	rpoB-F3	AATATGGAGGGTCGTCACGGTAACTAGGGT	330
	rpoB-R3	TTCAGCCAGAAGCAGAACTTCTTCATCGCT	
rpoB-Set 4	rpoB-F4	ATAAGATGGCGGGTCGTCACGGTCACAAGG	258
	rpoB-R4	CGTAAGCACGCTGGATGAATTCACGCAGTT	
rpoB-Set 5	rpoB-F5	CAGATCCTTGGAACTCACCTGGGTATGGCT	260
	rpoB-R5	TCTTTGATTTCACCTTCTTTCGCACCGTCG	
blaOXA48-Set 1	blaOXA48-F1	TTTCGATTATGGTAATGAGGACATTTCGGG	357
	blaOXA48-R1	TGTTTGAGCACTTCTTTTGTGATGGCTTGG	
blaOXA48-Set 2	blaOXA48-F2	TGCGGTAGCAAAGGAATGGCAAGAAAACAA	205
	blaOXA48-R2	CTTAACCACGCCCAAATCGAGGGCGATCAA	
blaOXA48-Set 3	blaOXA48-F3	GCGCAGCCAGCGTATTGTCAAACAAGCCAT	137
	blaOXA48-R3	TATCATCAAGTTCAACCCAACCGACCCACC	
blaOXA48-Set 4	blaOXA48-F4	CGGTAGCAAAGGAATGGCAAGAAAACAAAA	203
	blaOXA48-R4	CTTAACCACGCCCAAATCGAGGGCGATCAA	
blaOXA48-Set 5	blaOXA48-F5	GCCTTATCGGCTGTGTTTTTGGTGGCATCG	156
	blaOXA48-R5	GCTTGGTTCGCCCGTTTAATATTATTGGTA	

F, Forward primer; R, Reverse primer; _, mismatch.

To optimize RPA reaction conditions, we modified the reaction time and temperature. The reaction temperature was controlled at 37 C, and the reaction time was set to 15, 20, 25, 30, and 35 minutes, respectively (**Figures 2B**, **3B**, respectively). Because RPA reacts quickly, the interval of 5 minutes is chosen to avoid missing the best response time due to the excessive time interval (Lobato and O'Sullivan, 2018; Monteiro Belo Dos Santos et al., 2026). The results showed that there were fewer amplification products at 15 minutes, and the amplification products increased significantly in the 15–25 minute interval. After 25 minutes, the band intensity tended to stabilize. By fixing the reaction time at 30min, RPA amplifications were performed at 31, 34, 37, 40, and 43 C (**Figures 2C**, **3C**, respectively) (Lobato and O'Sullivan, 2018; Ji et al., 2023). Clear bands were obtained within the temperature range of 37-40 C. The amplification efficiency decreases at 43 C. Considering both time and economic costs, the optimal RPA amplification conditions were determined to be 37 C for 25 minutes, which was used in subsequent experiments. In addition, we optimized the primer concentration. The results indicate that as the primer concentration decreases, the content of RPA products gradually decreases. Therefore, 10 μM was chosen as the optimal primer concentration (**Figures 2D**, **3D**, respectively).

To evaluate whether RPA primers could specifically amplify target genes, seven clinically common pathogenic bacteria were used for RPA detection. Genomic DNA of these strains was adjusted to 100 ng/μL. As shown in **Figures 2D**, **3D**, no positive bands were observed when using genomic DNA from other pathogens as templates. The results indicated that the designed RPA primers exhibited good specificity and no cross-reactivity with other pathogenic bacteria.

### 3.2 Verification of Cas12a protein cleavage activity and optimization of CRISPR/Cas12a reaction conditions

Using the CRISPR RGEN Tools online design platform (http://www.rgenome.net/cas-designer/), specific crRNA sequences were successfully obtained, which exhibited an Out-of-frame Score > 65. The crRNA consists of a constantly repeated sequence structure linked to a spacer sequence complementary to the target sequence, as observed in the positive RPA amplicons. Specifically, the crRNA sequence for the *rpoB* gene was: UAAUUUCUACUAAGUGUAGAUAAGGAUCUGACCGAUGUUCAU. The crRNA sequence for the *blaOXA-48* gene was as follows: UAAUUUCUACUAAGUGUAGAUUGUUCAGUAAAGUGAGCAUUC. They include repeat sequences and spacer sequences targeting RPA amplification products. Wherein, “UAAUUUCUACUAAGUGUAGAU” is repeat sequence.

**Figure 4:**
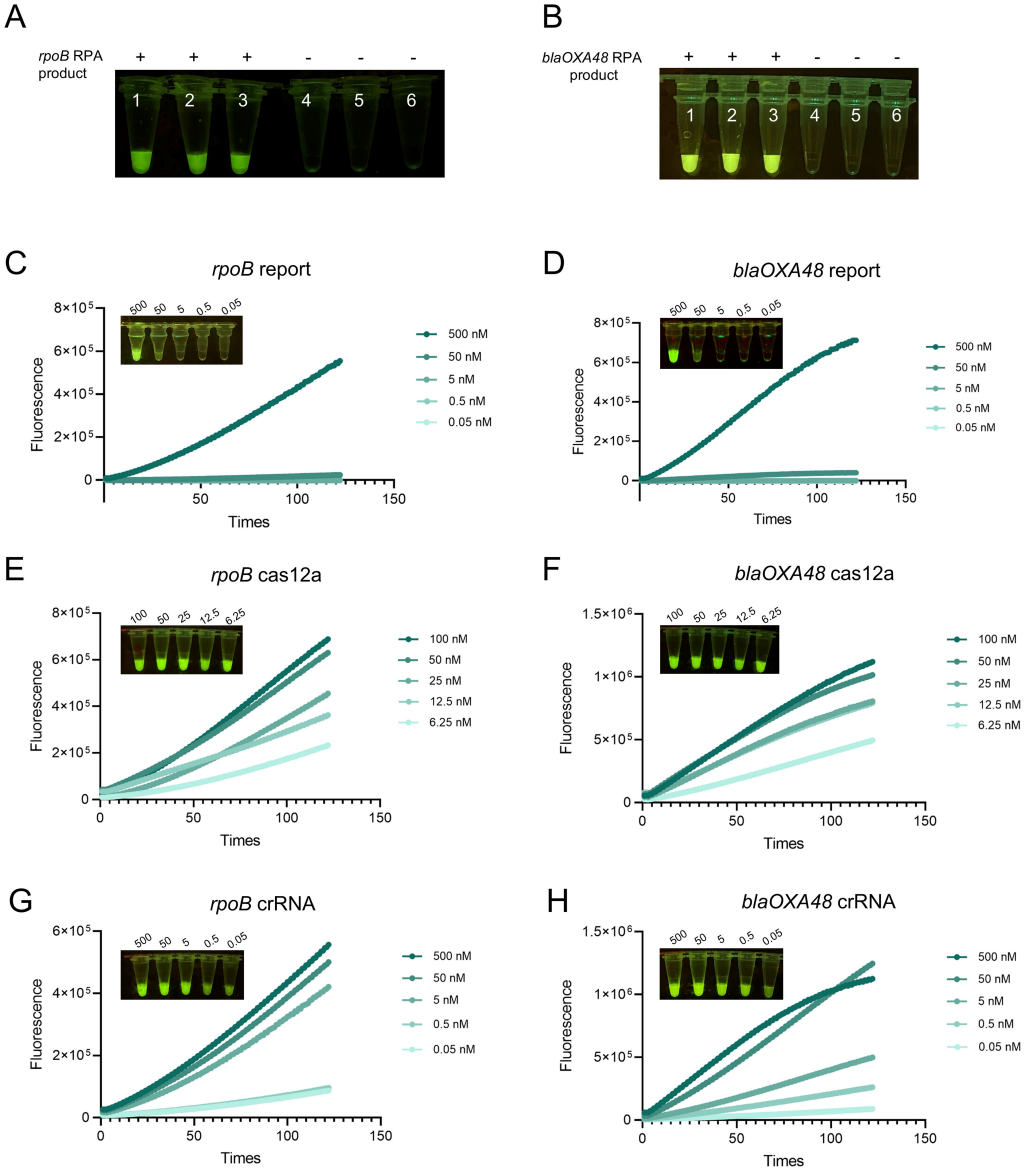
Verification of Cas12a protein cleavage activity and optimization of reaction conditions. **(A)** Verification results of Cas12a cleavage activity in the KP detection system. **(B)** Verification results of Cas12a cleavage activity in the CRKP detection system. **(C–H)**. Concentration optimization results for ssDNA, Cas12a, and crRNA in the RPA-CRISPR/Cas12a detection system. The fluorescence images were captured using a smartphone.

Two reaction systems were set up, each containing six reaction tubes. In the first group, tubes 1–3 were added with CRISPR/Cas12a reaction components and *rpoB*-specific RPA products, while tubes 4–6 lacked *rpoB* RPA products (other components were identical). In the second group, tubes 1–3 were added with CRISPR/Cas12a components and *blaOXA-48*-specific RPA products, and tubes 4–6 lacked *blaOXA-48* RPA products. After incubating both groups in a water bath at 37 C for 60 minutes, reaction products were visually detected using a fluorescence illuminator. Results showed significant fluorescent signals only in tubes with corresponding RPA amplicons, while no fluorescence was observed in tubes without RPA products (**Figures 4A, B**). These results indicate that Cas12a protein exhibits cleavage activity exclusively toward RPA amplicons containing specific target sequences within the CRISPR/Cas12a system, verifying the high specificity of its cleavage function.

To optimize the concentrations of components in the CRISPR/Cas12a reaction system, a series of concentration gradient experiments was conducted. Results showed that the fluorescence intensity was significantly higher at a probe concentration of 500 nM compared to other groups (**Figures 4C, D**). Cas12a protein concentration was positively correlated with fluorescence intensity, with no statistical difference between the 50 nM and 100 nM groups (**Figures 4E, F**). Increasing crRNA concentration also enhanced the fluorescent signal, and no significant difference was observed between the 50 nM and 500 nM groups (**Figures 4G, H**). Balancing detection sensitivity and economic cost, the optimal working concentrations were determined as 500 nM for the probe, 50 nM for Cas12a protein, and 50 nM for crRNA.

### 3.3 Evaluation of detection specificity and sensitivity of the CRISPR/Cas12a system

**Figure 5:**
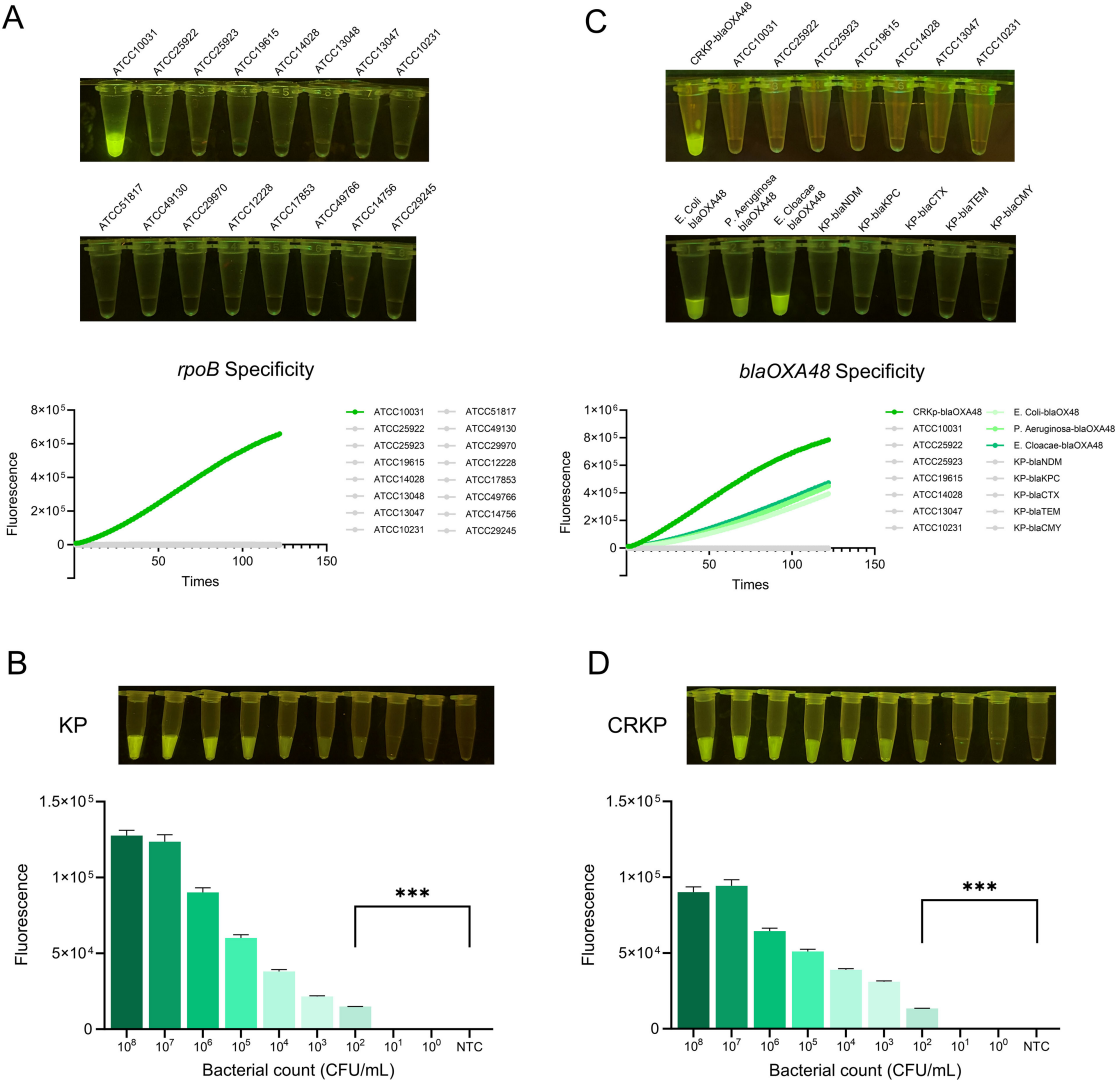
Evaluation of the specificity and sensitivity of the CRISPR/Cas12a assay. **(A)** Specificity validation of the *rpoB*-targeted CRISPR/Cas12a assay using other pathogenic microbial strains. **(B)** Sensitivity validation of CRISPR/Cas12a system for detecting KP. **(C)** Specificity validation of the *blaOXA-48*-targeted CRISPR/Cas12a assay using other standard pathogenic microbial strains and strains carrying other resistance genes. **(D)** Sensitivity validation of CRISPR/Cas12a system for detecting CRKP. The data was derived from three technical replicates, with bars representing the mean ± SEM. NTC, no template control. One-Way ANOVA was used to compare the fluorescence value across different groups, ***P<0.001.

To assess whether the CRISPR/Cas12a detection assay can specifically identify KP and CRKP harboring the *blaOXA-48* gene, this system was employed to detect other clinically prevalent pathogenic microorganisms. Fluorescence signals were visualized under blue light illumination, and prominent fluorescent signals were exclusively detected in reaction tubes containing KP or strains carrying the *blaOXA-48* gene (**Figures 5A, C**). The results indicated that the established RPA-CRISPR/Cas12a system for detecting KP and *blaOXA-48* gene exhibited excellent specificity, with no cross-reactivity with other pathogens. In addition, the two-step RPA-CRISPR/Cas12a assay was performed on bacterial suspensions of varying concentrations, with endpoint fluorescent signals monitored. The results showed that when the concentrations of KP and CRKP exceeded 10^2^ CFU/mL, the fluorescent signals were significantly enhanced (**Figures 5B, D**).

### 3.4 Construction of the one-tube RPA-CRISPR/Cas12a system

**Figure 6:**
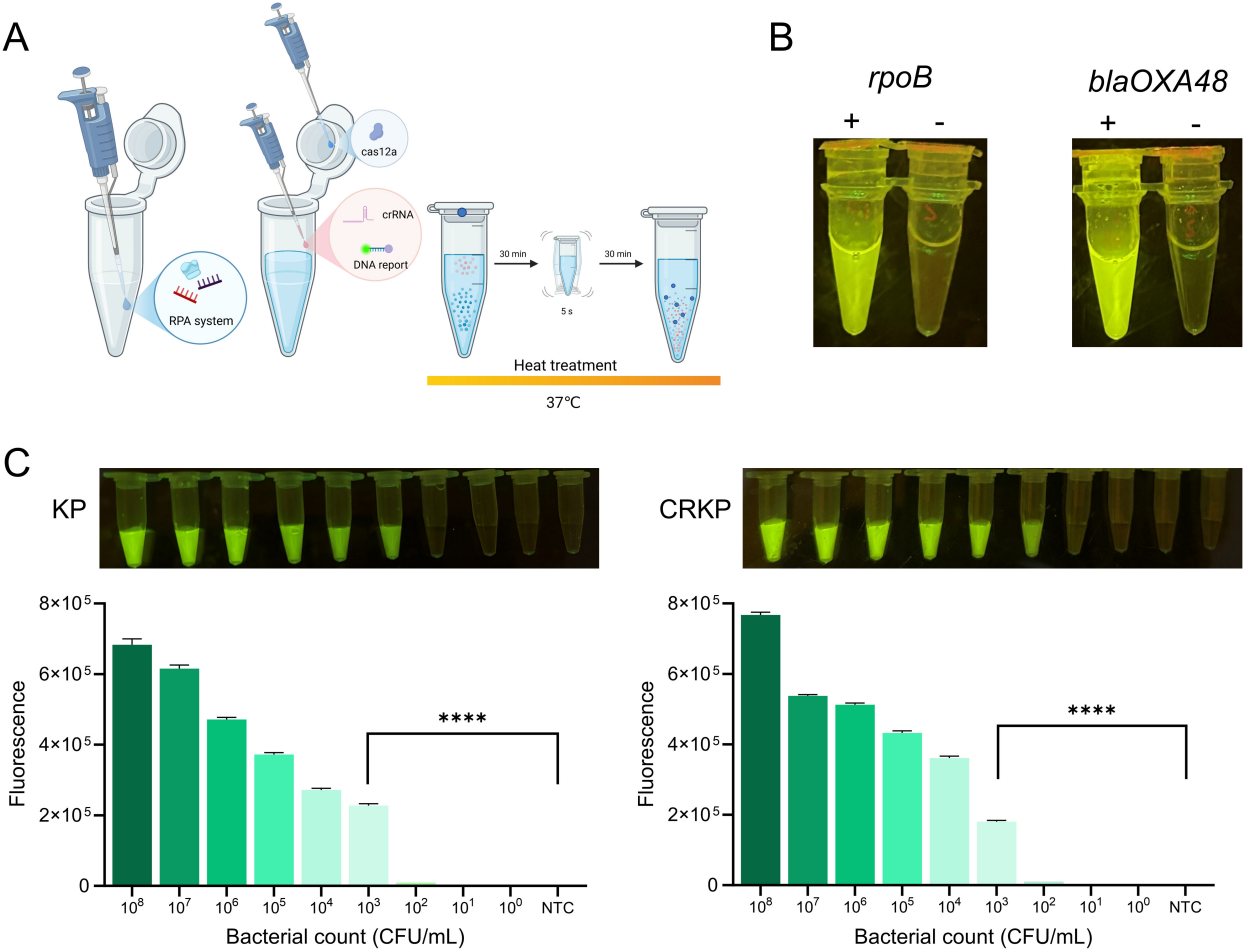
Construction of one-tube RPA-CRISPR/Cas12a system. **(A)** Schematic diagram of the construction method for one-tube RPA-CRISPR/Cas12a system. **(B)** Validation of one-tube RPA-CRISPR/Cas12a system for detecting KP and CRKP. **(C)** Sensitivity of one-tube RPA-CRISPR/Cas12a system for detecting KP and CRKP. The data was derived from three technical replicates, with bars representing the mean ± SEM. NTC, no template control. One-Way ANOVA was used to compare the fluorescence value across different groups, ****P<0.0001.

The one-tube RPA-CRISPR/Cas12a system is illustrated in the **Figure 6A**. When KP genome was added to the reaction tube containing *rpoB* primers, and *blaOXA-48*-carrying CRKP genome was added to the tube with *blaOXA-48* primers, both tubes showed positive results (**Figure 6B**). The one-tube RPA-CRISPR/Cas12a system was applied to detect bacterial suspensions at different concentrations, with fluorescence signals monitored in real time. Fluorescence significantly increased when KP and CRKP concentrations exceeded 10^3^ CFU/mL (**Figure 6C**).

**Figure 7:**
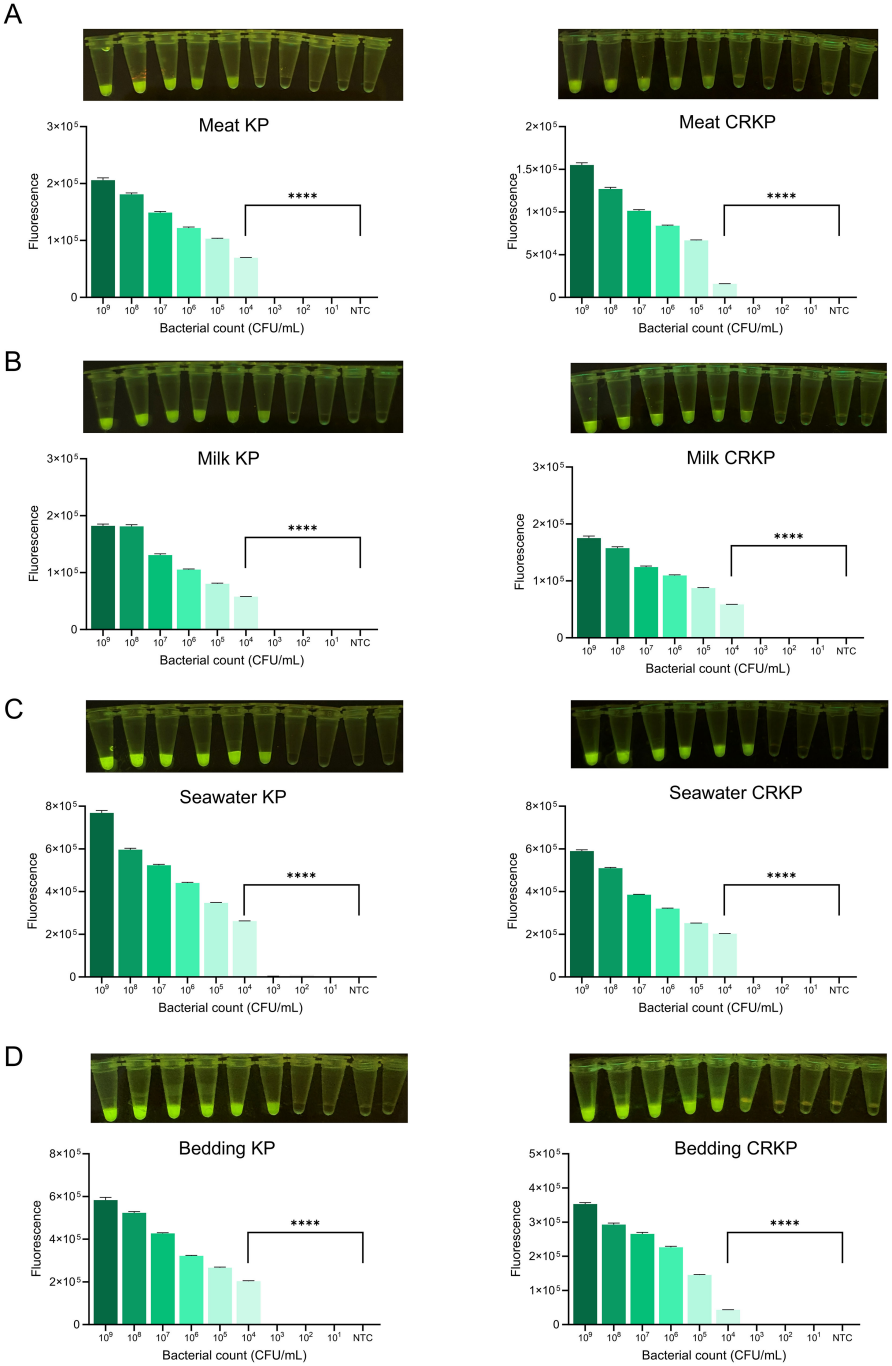
Sensitivity of one-tube RPA-CRISPR/Cas12a system for real samples. Sensitivity of one-tube RPA-CRISPR/Cas12a system for detecting KP and CRKP in real samples. The data was derived from three technical replicates, with bars representing the mean ± SEM. NTC, no template control; One-Way ANOVA was used to compare the fluorescence value across different groups, ****P<0.0001.

To evaluate the detection efficacy of the one-tube RPA-CRISPR/Cas12a system for real samples, we prepared solutions of meat, milk, seawater, and bedding materials containing different bacterial concentrations and performed detections using this system. The results showed that as the concentrations of KP and CRKP in the samples decreased, the fluorescence signal exhibited a gradient-decreasing trend. When the bacterial concentration exceeded 10^4^ CFU/mL, the fluorescence signal was significantly enhanced, showing a significant statistical difference compared with the no-template control group (**Figure 7**). Compared to the standard bacterial suspension samples, the overall fluorescence values were relatively lower when the system was used to detect real samples, and this was particularly evident in meat and milk samples. Given that the main components of milk and meat are proteins and fats, they may interfere with DNA extraction, the RPA amplification process, and the activity of Cas12a, thereby affecting the detection sensitivity. This result suggests that the components of complex matrices may impact the detection performance of this system. In subsequent work, it is necessary to optimize the pretreatment procedures to improve the detection effectiveness for real samples.

### 3.5 Application validation of the one-tube RPA-CRISPR/Cas12a system

**Figure 8:**
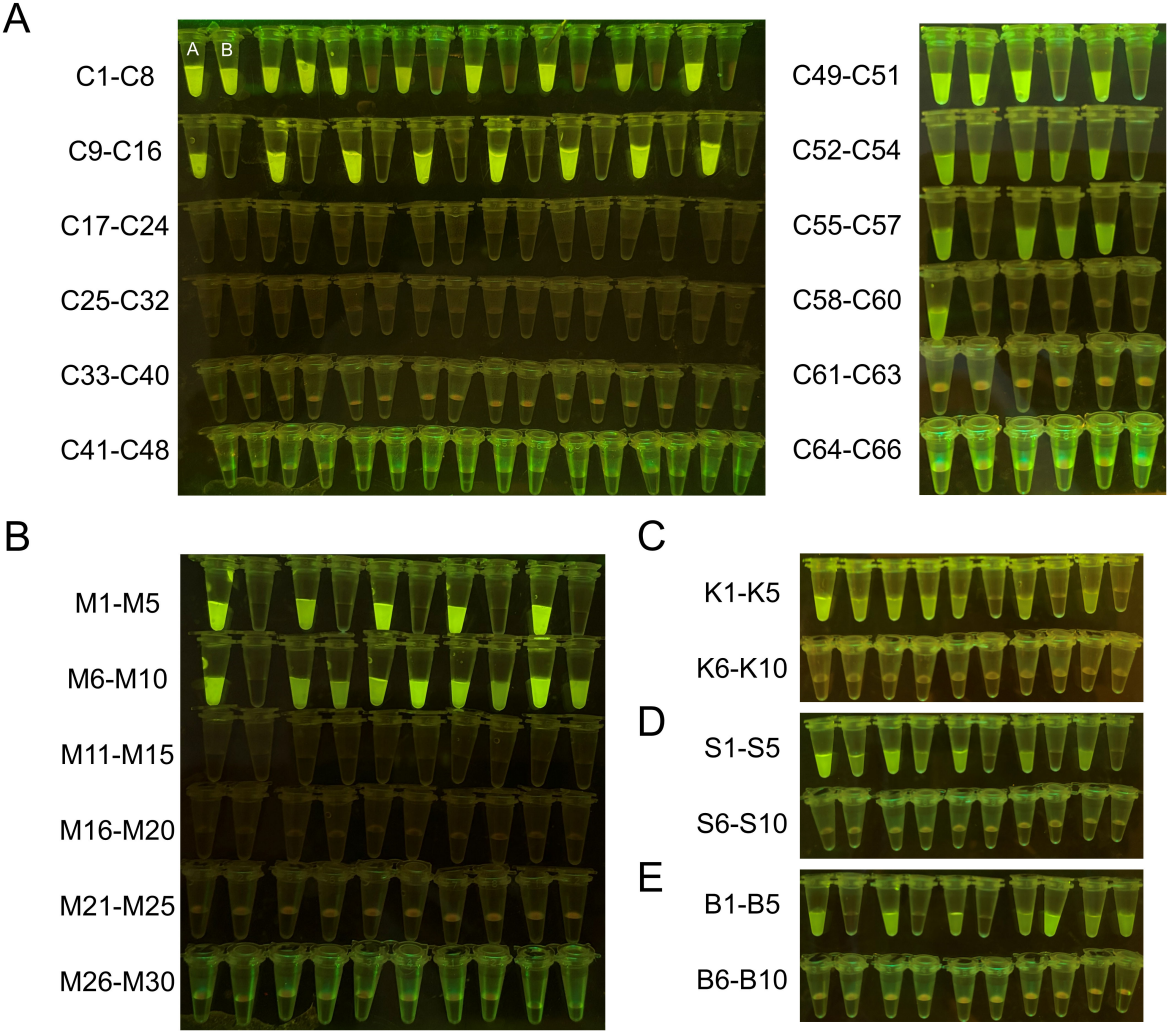
Application of one-tube RPA-CRISPR/Cas12a system. **(A)** Clinical application validation of one-tube RPA-CRISPR/Cas12a system (In tube A, the RPA-CRISPR/Cas12a reaction system targeting *rpoB* was added, while in tube B, the RPA-CRISPR/Cas12a reaction system targeting *blaOXA48* was incorporated). **(B)** Application validation of one-tube RPA-CRISPR/Cas12a system in meat samples. **(C)** Application validation of one-tube RPA-CRISPR/Cas12a system in milk samples. **(D)** Application validation of one-tube RPA-CRISPR/Cas12a system in seawater samples. **(E)** Application validation of one-tube RPA-CRISPR/Cas12a system in mouse bedding samples.

To comprehensively assess the clinical performance of the one-tube RPA-CRISPR/Cas12a detection system, a total of 66 clinical specimens were collected. In this dual-target assay, the RPA-CRISPR/Cas12a reaction mixture targeting the *rpoB* gene, a specific marker for KP, was loaded into Tube A, while the reaction mixture targeting the *blaOXA-48* gene, a key determinant of carbapenem resistance, was added to Tube B. The result interpretation criteria were rigorously defined as follows: positive detection of both fluorescence signals in Tubes A and B indicated co-infection with *blaOXA-48*-harboring CRKP; fluorescence solely in Tube A and absence of signal in Tube B were indicative of non-resistant KP infection; lack of fluorescence in Tube A but positive signal in Tube B suggested infection with non-KP bacteria carrying the *blaOXA-48* gene; finally, negative fluorescence signals in both tubes confirmed the absence of KP infection. Detection results showed that 26 samples were positive for KP and 6 were positive for *blaOXA-48*-carrying CRKP, as indicated by fluorescence signals and visual detection under blue light illumination (**Figure 8A**). The performance of RPA-CRISPR/Cas12a was compared with qPCR and traditional culture methods (**Table 2**). These results were consistent with those obtained by microbial culture and qPCR. The detection limit of qPCR method is 100 CFU/mL (**Supplementary Figure 4**), which was comparable to that of our newly developed RPA-CRISPR/Cas12a assay. These results revealed that the one-tube RPA-CRISPR/Cas12a assay exhibits 100% concordance with both microbial culture and qPCR for the detection of *rpoB* and the *blaOXA48* gene, respectively.

**Table 2:** Assay performance of different method.

Method	KP			CRKP-*blaOXA48*			Time of detection*
	Positive	Negative	Total	Positive	Negative	Total	
RPA-CRISPR/Cas12a	51	75	126	16	110	126	~60 min
qPCR	–	–	–	16	110	126	~120 min
Culture method	51	75	126	–	–	–	2 days

*It refers to entire detection time from extraction of DNA to the final determination.

Meanwhile, the one-tube RPA⁃CRISPR/Cas12a system was applied to detect food samples. In meat samples, ten samples were positive for KP, among which 4 were *blaOXA48*-type CRKP (**Figure 8B**). In the milk samples, 5 were positive for KP, among which 2 were identified as *blaOXA48*-type CRKP (**Figure 8C**). In the seawater samples, 5 were positive for KP, with 2 being *blaOXA48*-type CRKP (**Figure 8D**). Regarding the bedding samples, 5 were positive for KP, and 2 of these were *blaOXA48*-type CRKP (**Figure 8E**). The results were consistent with those of the microbial culture method and the qPCR method.

## 4 Discussion

As an important opportunistic pathogen, KP can cause various diseases such as pneumonia, bloodstream infections, urinary tract infections, and abdominal infections, particularly posing a significant threat to immunocompromised populations. As a drug-resistant strain of KP, CRKP has become a daunting challenge in clinical anti-infection therapy due to its high tolerance to carbapenem antibiotics (Yang et al., 2022). The complex resistance mechanisms of CRKP not only severely limit the clinical application of carbapenem drugs but pose severe challenges to infection control and patient prognosis, urgently demanding the development of new detection and treatment strategies (Jin et al., 2021). Current detection methods for CRKP mainly include traditional methods such as antimicrobial susceptibility testing, molecular biology methods like polymerase chain reaction, and mass spectrometry (Alsharksi et al., 2024). Although these methods can effectively detect CRKP, they rely on sophisticated and expensive equipment, entail cumbersome and time-consuming procedures, and require specialized expertise from operators (Liu et al., 2024). These drawbacks greatly restrict their popularization and application, especially in primary healthcare facilities and resource-constrained remote areas. Therefore, developing a rapid, convenient, low-cost CRKP detection technology is significant.

An ideal point-of-care testing tool should possess characteristics such as speed, precision, and user-friendliness (Fu et al., 2025). RPA enables the amplification of target nucleic acids under isothermal conditions with mild reaction conditions and simple instrument requirements, providing an ideal technical foundation for the development of POCT (Liu et al., 2023). However, RPA still has some shortcomings, such as false-positive results caused by improper primer design (Piepenburg et al., 2006). CRISPR/Cas12a can accurately recognize and bind to target DNA sequences, activating the trans-cleavage activity of Cas12a to cleave surrounding single-stranded DNA non-specifically (Wang et al., 2023). Notably, this recognition process is highly dependent on the complementary pairing of crRNA with the target sequence, and mismatches at different sites of crRNA can affect the cleavage activity of Cas12a (Strohkendl et al., 2018; Lin et al., 2026). The combination of RPA and CRISPR/Cas12a systems enables rapid detection of pathogenic microorganisms. The advantage of RPA’s rapid isothermal amplification is that it meets the CRISPR/Cas system requirement for the number of detection targets. Meanwhile, CRISPR/Cas12a possesses high specificity and powerful signal amplification functions, which can solve the non-specificity problem of RPA. The combination of the two significantly enhances the accuracy, convenience, and applicability of detection, especially enabling instant and precise diagnosis in resource-constrained remote areas (Li et al., 2023). Recently, a light–controlled RPA–CRISPR/Cas12a system targeting the *rcsA* gene was developed for rapid detection of KP (Pan et al., 2025). Another RPA–CRISPR/Cas12a platform validated the reliability of this combined strategy in clinical settings (Tan et al., 2024). However, these previous methods focused only on single–target pathogen identification and did not include the critical carbapenem resistance gene. In comparison, our dual–target one-tube system allows simultaneous detection of KP and *blaOXA-48*–harboring CRKP, with closed–tube anti–contamination and blue–light visual readout, making it more suitable for clinical diagnosis and food safety surveillance.

This study selected the intrinsic gene *rpoB* and the carbapenem resistance gene *blaOXA-48* as detection targets. The *rpoB* gene is an intrinsic gene of KP (Patole et al., 2021), widely present in KP, and primarily responsible for encoding the β-subunit of RNA polymerase, a key gene in the survival and transcription processes of KP. Using the *rpoB* gene as a detection target enables precise identification of KP (Moter et al., 2013). The *blaOXA-48* gene is a carbapenem resistance gene encoding the *blaOXA-48*-type β-lactamase (Berger et al., 2013). This enzyme specifically recognizes the β-lactam ring in carbapenem antibiotics and performs a nucleophilic attack on the β-lactam ring via a serine residue at the active site. This opens the ring and inactivates carbapenem antibiotics, thus leading to bacterial drug resistance. In addition, the detection rate of *blaOXA-48* was high in Lianyungang area. Therefore, the detection of the *blaOXA-48* gene is of significant importance for clinical precision diagnosis and rational use of antimicrobial agents.

In this study, the dual-target detection strategy can distinguish KP and CRKP, which addresses the limitation of the single gene detection method (Tan et al., 2024; Fu et al., 2025; Pan et al., 2025). Additionally, two-step and one-tube RPA-CRISPR/Cas12a systems were developed, with their detection performances comparatively analyzed. Results indicated that the single-tube assay exhibiting approximately one order of magnitude lower sensitivity. This observation may be attributed to potential mutual interference between reaction components: CRISPR-related reagents might compromise RPA amplification efficiency, while RPA components could inhibit Cas12a enzymatic activity, thereby reducing its cleavage efficiency. Although the one-tube RPA-CRISPR/Cas12a system constructed in this study has been improved and evaluated for real sample applicability, this method still has several limitations: 1) This method can only detect blaOXA-48-type KP and cannot distinguish other drug-resistant genes, which limits the application range of this detection method. 2) The detection was performed only in the laboratory and showed good results in artificial pollution simulation samples. It is still necessary to expand the sample types in the follow-up study and to further conduct larger-scale clinical and on-site validation of naturally contaminated real food and environmental samples, so as to more comprehensively evaluate its practical application value. 3) The sensitivity is relatively low. Most existing studies evaluate the analytical performance of RPA-CRISPR/Cas12a for KP detection using plasmid or genomic DNA standards (Wu et al., 2025; Wei et al., 2026). In comparison, this study uses live bacteria counting method to carry out the evaluation, which is more suitable for the clinical actual scene, and has significant application advantages compared with the traditional evaluation method. In addition, the RPA-CRISPR/Cas12a method established by Zheng for detection of *Burkholderia gladioli* in food matrices, with a detection line as low as 10 CFU/mL (Zheng et al., 2023). Shen developed a rapid detection system for *Candida albicans* based on affinity-magnetic separation combined with the RPA-CRISPR/Cas12a method, with a detection sensitivity of 30 CFU/mL in blood and BALF (Shen et al., 2024). The detection sensitivity of the one-step RPA CRISPR/Cas12a system established in this study in a pure bacterial solution is comparable to levels reported in the literature. However, in clinical validation using real samples, the diagnostic sensitivity of our method is slightly lower than that reported by Shen et al. The reason for this difference may be Shen’s use of affinity magnetic separation for pre-enrichment, which effectively improves the capture efficiency of target fungi, whereas our method is a one-step reaction and does not introduce an independent enrichment step. However, our system is easier to operate, and is better suited to the rapid screening needs of grassroots and resource-limited settings. Future research should focus on further enhancing reaction sensitivity. Potential strategies to achieve this include selecting reference genes with high expression levels, as well as developing more efficient sample lysis buffers to optimize genome extraction efficiency. Combining the RPA-CRISPR/Cas12a system with visualization technologies and surface-enhanced Raman spectroscopy to fabricate novel sensing technologies may enhance sensitivity and accuracy. Furthermore, in future studies, betaine, polyethylene glycol, and dimethyl sulfoxide could be considered for addition to the RPA reaction system to reduce the formation of DNA secondary structures, facilitate DNA strand separation, and improve amplification efficiency (Yang et al., 2023; Wang et al., 2025). 4) After the reaction, blue light irradiation is required to visually observe the fluorescence response, which increases equipment costs. In the follow-up, nanozyme materials can be combined with RPA-CRISPR/Cas12a to develop a colorimetric detection technology enabling visual observation under natural light (Arshad et al., 2024). Notably, the one-pot RPA-CRISPR/Cas12a strategy combined with an innovative electrochemical lateral flow strip (OPRCC-eLFS), has been reported for ultrasensitive and precise detection of Salmonella (Chen et al., 2025). Additionally, efforts should be dedicated to establishing high-throughput RPA-CRISPR/Cas12a technologies and developing integrated nucleic acid extraction-RPA reaction-detection instruments to facilitate the rational utilization of medical resources. Recently, a one-pot chip based on the RPA-CRCISP12a platform has been developed to detect nucleic acids from intestinal pathogens (Ren et al., 2024). The chip enables isothermal, high-throughput simultaneous detection of multiple samples with improved integration. The assay achieved excellent linearity over 10^2–^10^8^ CFU/mL. This portable and programmable platform shows great potential for early screening of gastrointestinal inflammatory diseases.

## 5 Conclusion

This study combined RPA and CRISPR/Cas12a technologies for the synchronous detection of KP and CRKP. In the two-step reaction, CRISPR/Cas12a system could detect 10^2^ CFU/mL of KP and CRKP. Furthermore, a one-tube RPA-CRISPR/Cas12a detection method was established. This one-tube platform enables rapid, visual, contamination-safe detection of KP and locally prevalent blaOXA-48-type CRKP within 1 hour. It prioritizes usability for POCT and food surveillance rather than ultrahigh sensitivity, providing a practical tool for resource-limited settings. In conclusion, this study offers a novel solution for rapid on-site detection of Klebsiella pneumoniae, demonstrating significant application value.

## Data Availability

The original contributions presented in the study are included in the article/**Supplementary Material**. Further inquiries can be directed to the corresponding author.
