# Supplementary material for: Visual detection platform based on RPA-CRISPR/Cas12a for Klebsiella pneumoniae and Carbapenem-resistant Klebsiella pneumoniae in clinical and food safety settings
Source: Front Cell Infect Microbiol. 2026 May 25;16:1817859. doi: 10.3389/fcimb.2026.1817859 (PMC13243421; doi:10.3389/fcimb.2026.1817859)
Supplement: Supplementary file 1 [file Table1.docx]

Supplementary Material

# Supplementary Methods

**Inclusion and Exclusion Criteria for Clinical Specimens**

**Inclusion Criteria**

Patient source: Patients for whom clinicians highly suspected bacterial infection (potentially Klebsiella pneumoniae infection) based on clinical symptoms or preliminary laboratory tests.

Specimen quality:

Sputum specimens must comply with the requirements specified in the Guidelines for Standardized Collection and Processing of Clinical Microbiology Test Specimens; Urine specimens must be clean midstream urine; Bronchoalveolar lavage fluid specimens must be collected by professional medical staff via bronchoscopic procedure, with strict aseptic technique throughout the collection process.

**Exclusion Criteria**

Specimens from patients who had received carbapenem antibiotics (e.g., imipenem, meropenem) or other broad-spectrum antibiotics (e.g., cephalosporins, quinolones) within 48 hours prior to specimen collection. Specimens that failed to meet the quality standards.

A total of 66 clinical specimens in this study were obtained through consecutive collection, following the specific procedure below: Within the study-defined time window, all specimens that met the aforementioned inclusion criteria and did not trigger any exclusion criterion were collected consecutively in accordance with the "first-come, first-collected" principle, without prior screening.

# Supplementary Tables

Table S1. Drug-resistant genotyping on CRKP strains clinically isolated from our hospital

| Patient number | Drug-resistant genotyping by qPCR |
| --- | --- |
| 1 | *blaOXA-48* |
| 2 | *blaNDM* |
| 3 | *blaNDM* |
| 4 | *blaOXA-48* |
| 5 | *blaKPC* |
| 6 | *blaOXA-48* |
| 7 | *blaKPC* |
| 8 | *blaKPC* |
| 9 | *blaKPC* |
| 10 | *blaOXA-48* |
| 11 | *blaKPC* |
| 12 | *blaOXA-48* |
| 13 | *blaOXA-48* |
| 14 | *blaKPC* |
| 15 | *blaKPC* |
| 16 | *blaKPC* |
| 17 | *blaOXA-48* |
| 18 | *blaOXA-48* |
| 19 | *blaKPC* |
| 20 | *blaOXA-48* |
| 21 | *blaKPC* |
| 22 | *blaKPC* |
| 23 | *blaKPC* |
| 24 | *blaNDM* |
| 25 | *blaOXA-48* |
| 26 | *blaNDM* |
| 27 | *blaOXA-48* |
| 28 | *blaOXA-48* |
| 29 | *blaNDM* |
| 30 | *blaOXA-48* |
| 31 | *blaKPC* |
| 32 | *blaOXA-48* |
| 33 | *blaKPC* |
| 34 | *blaNDM* |
| 35 | *blaNDM* |
| 36 | *blaKPC* |
| 37 | *blaOXA-48* |
| 38 | *blaOXA-48* |
| 39 | *blaNDM* |
| 40 | *blaOXA-48* |
| 41 | *blaOXA-48* |
| 42 | *blaKPC* |
| 43 | *blaNDM* |
| 44 | *blaNDM* |
| 45 | *blaOXA-48* |
| 46 | *blaKPC* |
| 47 | *blaKPC* |
| 48 | *blaNDM* |
| 49 | *blaOXA-48* |
| 50 | *blaOXA-48* |
| 51 | *blaKPC* |

Table S2. Information and identification of the clinical samples

| Number | Specimen | Result of culture method (KP) | Result of qPCR (CRKP) | Result of one-tube RPA-CRISPR/Cas12a (KP/CRKP) |
| --- | --- | --- | --- | --- |
| S1 | Sputum | Positive | *blaOXA-48* | Positive/ *blaOXA-48* |
| S2 | BALF | Positive | *blaOXA-48* | Positive/ *blaOXA-48* |
| S3 | Sputum | Positive | - | Positive/- |
| S4 | Sputum | Positive | - | Positive/- |
| S5 | Sputum | Positive | - | Positive/- |
| S6 | BALF | Positive | - | Positive/- |
| S7 | BALF | Positive | - | Positive/- |
| S8 | Sputum | Positive | - | Positive/- |
| S9 | Sputum | Positive | - | Positive/- |
| S10 | Sputum | Positive | - | Positive/- |
| S11 | Urine | Positive | - | Positive/- |
| S12 | Sputum | Positive | - | Positive/- |
| S13 | Sputum | Positive | - | Positive/- |
| S14 | Sputum | Positive | - | Positive/- |
| S15 | BALF | Positive | - | Positive/- |
| S16 | Sputum | Positive | - | Positive/- |
| S17 | BALF | Negative | - | Negative/- |
| S18 | Sputum | Negative | - | Negative/- |
| S19 | Sputum | Negative | - | Negative/- |
| S20 | Sputum | Negative | - | Negative/- |
| S21 | Sputum | Negative | - | Negative/- |
| S22 | Sputum | Negative | - | Negative/- |
| S23 | Sputum | Negative | - | Negative/- |
| S24 | Sputum | Negative | - | Negative/- |
| S25 | Sputum | Negative | - | Negative/- |
| S26 | Urine | Negative | - | Negative/- |
| S27 | Sputum | Negative | - | Negative/- |
| S28 | Urine | Negative | - | Negative/- |
| S29 | Sputum | Negative | - | Negative/- |
| S30 | Sputum | Negative | - | Negative/- |
| S31 | Sputum | Negative | - | Negative/- |
| S32 | Sputum | Negative | - | Negative/- |
| S33 | Sputum | Negative | - | Negative/- |
| S34 | Sputum | Negative | - | Negative/- |
| S35 | Sputum | Negative | - | Negative/- |
| S36 | Sputum | Negative | - | Negative/- |
| S37 | BALF | Negative | - | Negative/- |
| S38 | Sputum | Negative | - | Negative/- |
| S39 | Sputum | Negative | - | Negative/- |
| S40 | Sputum | Negative | - | Negative/- |
| S41 | BALF | Negative | - | Negative/- |
| S42 | Sputum | Negative | - | Negative/- |
| S43 | Urine | Negative | - | Negative/- |
| S44 | Sputum | Negative | - | Negative/- |
| S45 | Sputum | Negative | - | Negative/- |
| S46 | BALF | Negative | - | Negative/- |
| S47 | Urine | Negative | - | Negative/- |
| S48 | Sputum | Negative | - | Negative/- |
| S49 | Sputum | Positive | *blaOXA-48* | Positive/ *blaOXA-48* |
| S50 | Sputum | Positive | - | Positive/- |
| S51 | Sputum | Positive | - | Positive/- |
| S52 | Sputum | Positive | *blaOXA-48* | Positive/ *blaOXA-48* |
| S53 | Sputum | Positive | *blaOXA-48* | Positive/ *blaOXA-48* |
| S54 | Sputum | Positive | - | Positive/- |
| S55 | Sputum | Positive | - | Positive/- |
| S56 | Sputum | Positive | *blaOXA-48* | Positive/ *blaOXA-48* |
| S57 | Sputum | Positive | - | Positive/- |
| S58 | Sputum | Positive | - | Positive/- |
| S59 | Sputum | Negative | - | Negative/- |
| S60 | Sputum | Negative | - | Negative/- |
| S61 | Sputum | Negative | - | Negative/- |
| S62 | Sputum | Negative | - | Negative/- |
| S63 | Sputum | Negative | - | Negative/- |
| S64 | Sputum | Negative | - | Negative/- |
| S65 | Sputum | Negative | - | Negative/- |
| S66 | Sputum | Negative | - | Negative/- |

# Supplementary Figures


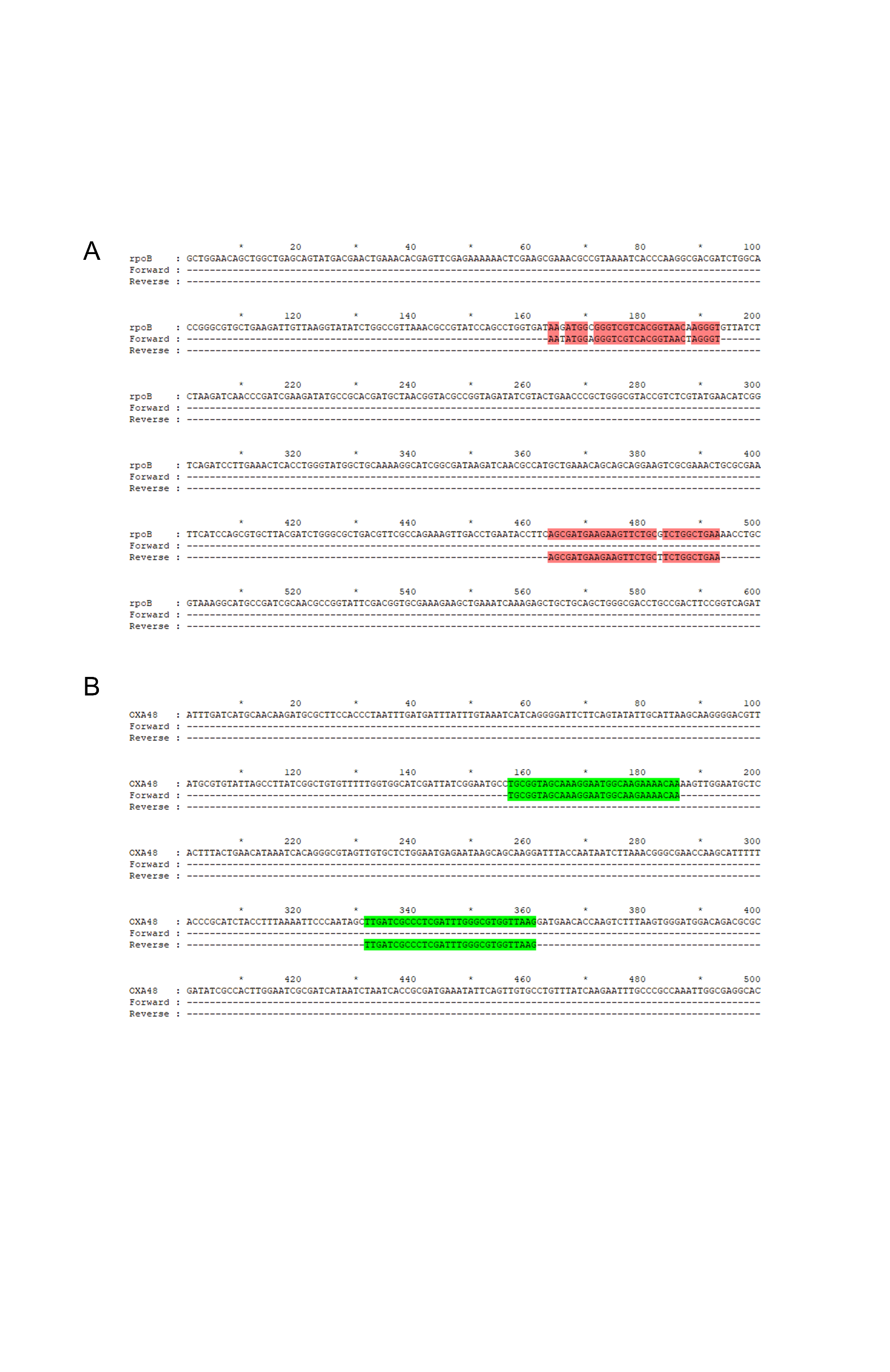


**Figure S1. Sequence alignment**

A. Sequence alignment of the *rpoB* gene and primers was performed using MEGA, and the sequence alignment figure was prepared using GeneDoc. B. Sequence alignment of the *blaOXA-48* gene and primers was performed using MEGA, and the sequence alignment figure was prepared using GeneDoc.


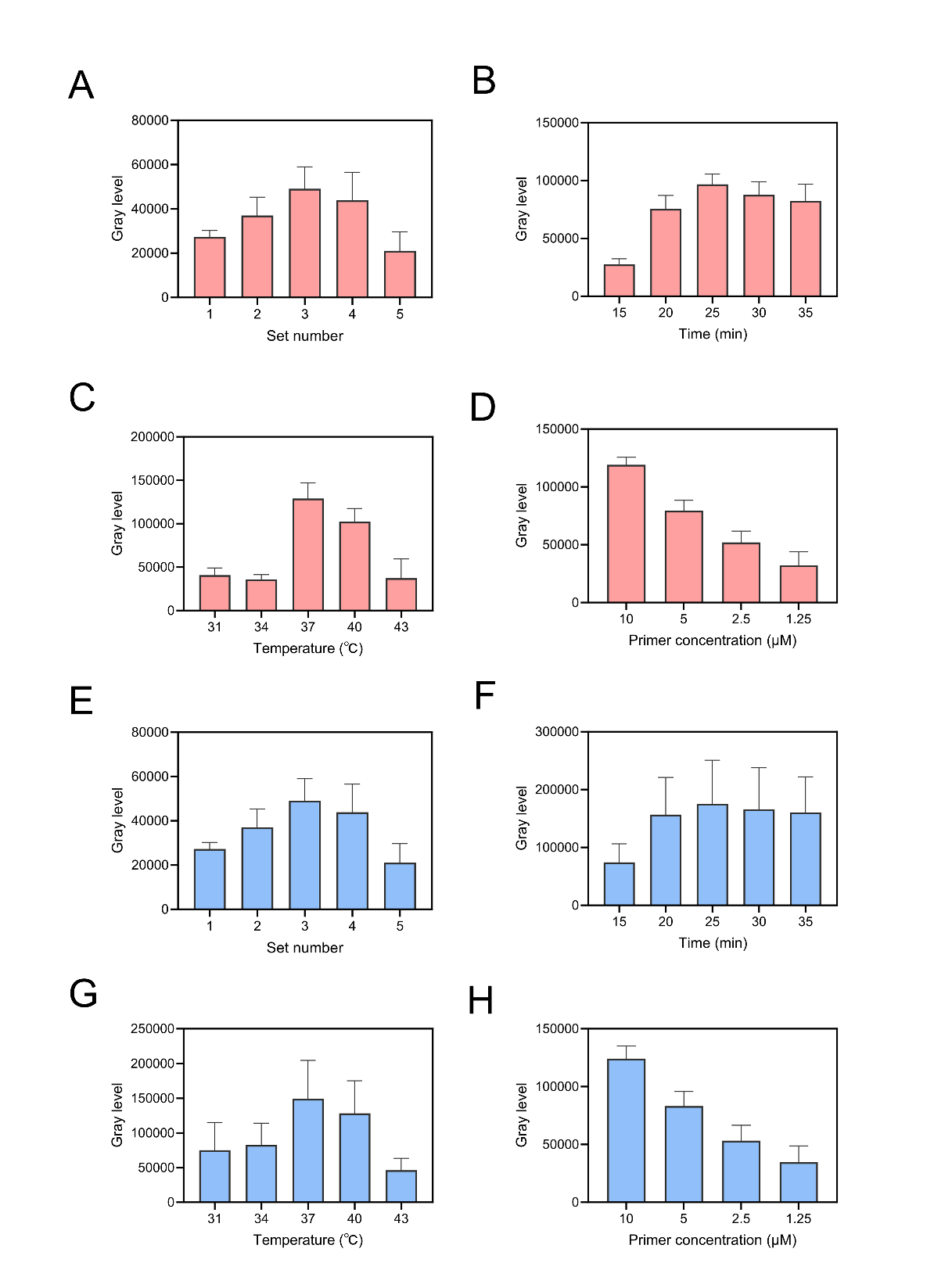


**Figure S2. Gray value statistics of agarose gel**

A. Gray value statistics of RPA primer screening for the *rpoB* gene. B. Gray value statistics of RPA reaction time optimization for the *rpoB* gene. C. Gray value statistics of RPA reaction temperature optimization for the *rpoB* gene. D. Gray value statistics of primer concentrations optimization for the *rpoB* gene. E. Gray value statistics of RPA primer screening for the *blaOXA-48* gene. F. Gray value statistics of RPA reaction time optimization for the *blaOXA-48* gene. G. Gray value statistics of RPA reaction temperature optimization for the *blaOXA-48* gene. H. Gray value statistics of primer concentrations optimization for the *blaOXA-48* gene.


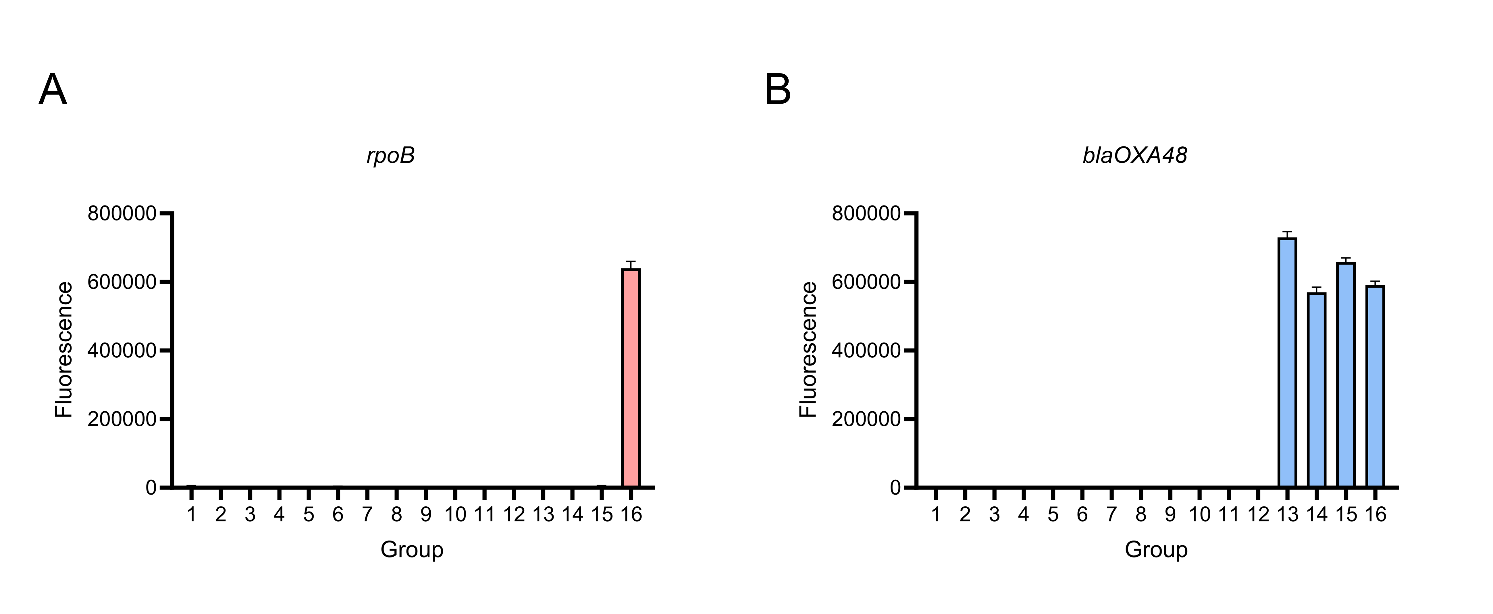


**Figure S3. CRISPR assay of RPA products generated from non-target templates**

A. CRISPR assay of RPA products of *rpoB* generated from non-target templates. Groups 1-15 were non-target templates, and group 16 was *Klebsiella pneumoniae* templates. B. CRISPR assay of RPA products of *blaOXA48* generated from non-target templates. Groups 1-12 were non-target templates, and groups 13-16 were *blaOXA48* carbapenem resistant pathogen templates.


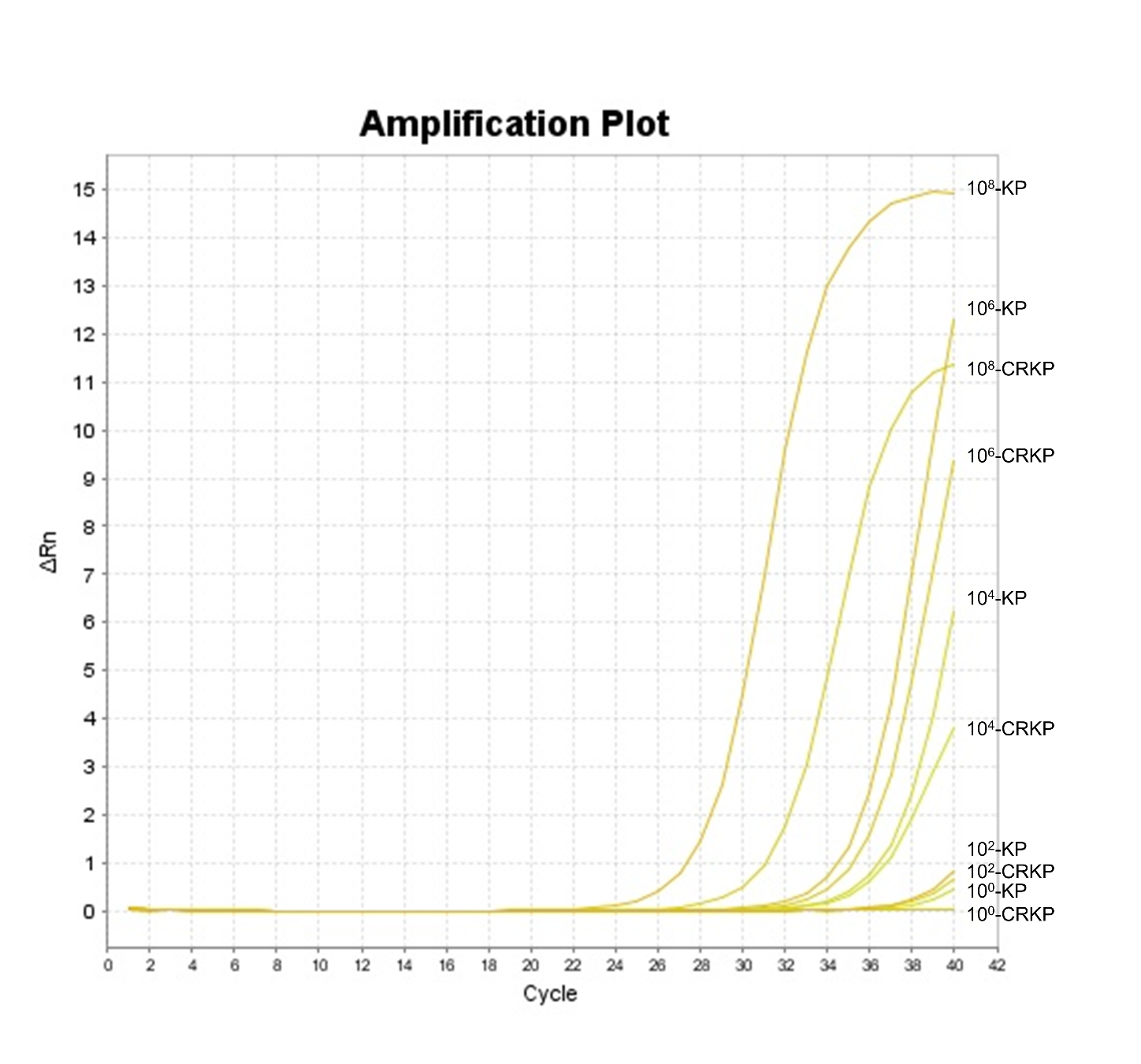


**Figure S4. The detection limit of qPCR method**
